# Limited Impact of Serial Follow-Up Imaging in Clinically Stable Patients With Brainstem Cavernous Malformations

**DOI:** 10.3389/fneur.2020.00789

**Published:** 2020-08-06

**Authors:** Julia Velz, Flavio Vasella, Yang Yang, Marian Christoph Neidert, Luca Regli, Oliver Bozinov

**Affiliations:** ^1^Department of Neurosurgery, University Hospital Zurich, Zurich, Switzerland; ^2^Clinical Neuroscience Center, University of Zurich, Zurich, Switzerland

**Keywords:** brainstem cavernous malformations, BSCM, cerebral cavernous malformations, imaging, follow-up

## Abstract

**Background:** Clinical management of patients with brainstem cavernous malformations (BSCM) is often challenging due to the unpredictable clinical course and lack of high-quality evidence. Nevertheless, radiologic follow-up is often performed routinely. The objective of this work was to investigate whether active follow-up by serial imaging is justified and how planned imaging will impact clinical decision making in absence of clinical progression.

**Methods:** We included all consecutive patients with BSCM treated and followed at our Department between 2006 and 2018.

**Results:** Of 429 patients with CCM, 118 were diagnosed with BSCM (27.5%). Patients were followed for a mean of 8.1 (± 7.4 SD) years. Conservative treatment was recommended in 54 patients over the complete follow-up period, whereas 64 patients underwent surgical extirpation of BSCM. In total, 75 surgical procedures were performed. Over a period of 961 follow-up years in total, routinely performed follow-up MRI in clinically stable patients did not lead to a single indication for surgery.

**Conclusion:** Due to the difficult-to-predict clinical course of patients with BSCM and the relatively high risk associated with surgery, routine imaging is unlikely to have any influence on surgical decision making in clinically stable patients with BSCM.

## Introduction

Cerebral cavernous malformations (CCM) are among the most common vascular lesions of the central nervous system (CNS). While a majority of CCM are located supratentorially, a significant subset comprising ~15–18% of all intracranial CCM affect the brainstem (1). The clinical management of brainstem cavernous malformations (BSCM) poses a particular challenge. Given the highly eloquent anatomy with high density of cranial nerve nuclei and fiber tracts within the brainstem, even small BSCM hemorrhages can cause severe neurological symptoms. Furthermore, BSCM are associated with an increased risk of hemorrhage compared to CCM in other locations (1–3). It is unknown whether this is due to the fact that CCM hemorrhages that cause significant symptomatology in the brainstem would go unnoticed in an ineloquent supratentorial location, implying that supratentorial CCM hemorrhages are underdiagnosed relative to those in the brainstem. In any case, it is known that especially recurrent BSCM hemorrhages often create a pattern of progressive neurological decline, with most patients never returning to their prehemorrhage baseline (2, 4).

Risk assessment with regard to morbidity and mortality is essential to balance the risk of surgery against the natural history of BSCM when deciding whether or not to offer surgery. The decision to recommend surgery is difficult and depends on a number of factors such as patient presentation, lesion size, accessibility, distance to the pial or ependymal surface, history of repetitive hemorrhage and surgeon expertise, among others. However, assessing and discussing the indication for surgical treatment of BSCM is beyond the scope of the manuscript (1–3).

In cases of unclear distinction of BSCM from that of BSCM mimics due to initial acute brainstem hemorrhage, follow-up MRI within 3 months is recommended to confirm the diagnosis of BSCM (5–7).

As the clinical course of patients with BSCM is unpredictable and high-quality evidence is lacking, it is unclear whether or not active, serial clinical and radiological follow-up should be recommended in the further course. Currently, indication and timing of follow-up imaging in patients with BSCM is likely based on individual judgment.

Given the lack of literature on follow-up strategies for patients with BSCM (8), the objective of this study was to critically address whether serial follow-up imaging in clinically stable patients with BSCM is justified and how frequently imaging will influence clinical decision making in these cases.

## Materials and Methods

### Study Design and Patient Population

The present work is an observational, retrospective single-center study encompassing all patients with CCM that presented to our institution between 2006 and 2018. Inclusion criteria were as follows: Radiological or histological diagnosis of BSCM (1) and availability of follow-up data (2). Diagnosis of BSCM was confirmed by a board-certified neuroradiologist on imaging, and histologically by a board-certified neuropathologist when tissue was available for analysis.

### Data Collection

The medical history of each patient was analyzed for age, gender and clinical symptoms. In addition, date of first diagnosis, date of first contact to any neurosurgical department, date of last follow-up, as well as the number and dates of MRI-studies were collected. MRI-studies always comprised at least T1-weighted imaging (with and without contrast enhancement), T2-weighted imaging, and gradient echo sequences in all three planes (axial, coronal and sagittal) or susceptibility-weighted imaging (SWI). The dates and indications for each surgical intervention were obtained, as was the type of patient contact—being elective (outpatient consultation), referral to our institution for second opinion, or emergency (in-patient consultation/emergency room).

### Patient Management

At our department, we tend to recommend conservative management to patients with BCSM in absence of severe neurological deficits, and we restrain from operating after the first hemorrhage or when spontaneously improving neurological deficits occur. In cases of recurrent BSCM hemorrhage surgical treatment is further evaluated and discussed.

We perform routine radiological and clinical follow-up 6 months after initial diagnosis of BSCM and yearly in patients with known BSCM, comprising both MRI studies and consultation with a specialized neurosurgeon.

### Statistical Analysis

Descriptive statistical analysis was performed with IBM SPSS Statistics version 20.0 (IBM Corp., Armonk, New York, USA).

## Results

### Study Population and Patient Demographics

**Figure 1:**
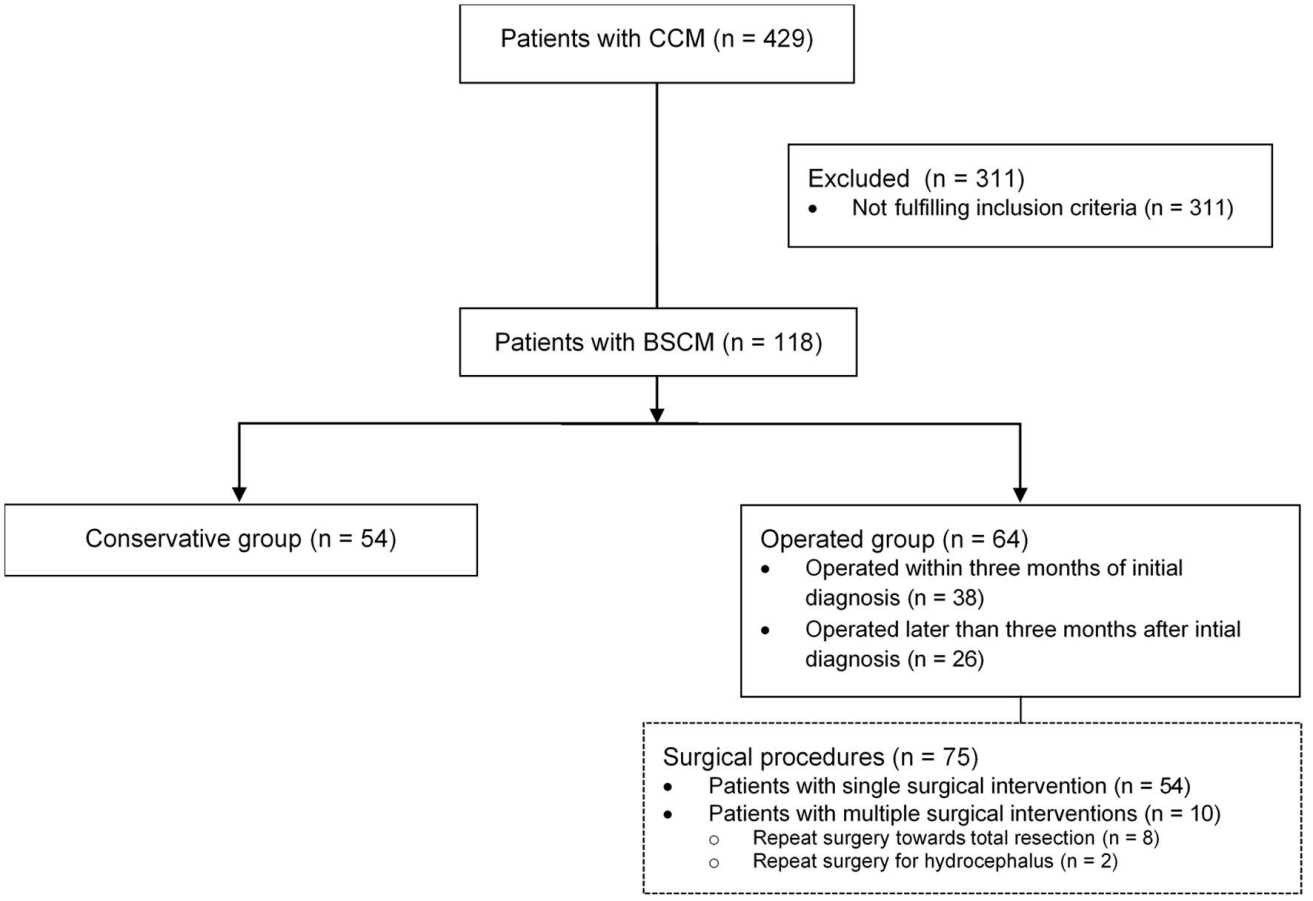
Analyzed patient cohort: From 429 patients with CCM, *n* = 118 (27.5%) patients harbored BSCM; in *n* = 64 surgical treatment was performed, whereas in *n* = 54 patients conservative treatment was performed. Seventy-five surgical interventions were performed in total.

Four hundred and twenty-nine patients with CCM were seen at our institution between 2006 and 2018. Of those, *n* = 118 (27.5%) patients harbored BSCM, fulfilled the above-mentioned criteria for this study and were included in the analysis (Figure 1). The mean age at first diagnosis was 41.1 years (± 17.11 SD; range 7.46–84.64); 44.9% of the patients were male (Table 1). The mean follow-up was 8.1 (±7.4 SD) years per patient. The total follow-up time was 961 person-years.

**Table 1:** Baseline patient characteristics.

**Age in years (mean ± SE)**	**41.1**	**± 17.11**
	No. of patients (Total number 118)	%
Sex		
Male Female	53 65	44.9% 55.1%
Symptomatic Asymptomatic	17 101	14.4% 85.6%
Conservative treatment Surgical treatment • Within 3 months after initial diagnosis • During Follow-Up	54 64 38 26	45.8% 54.2%
Indication for surgical treatment based on		
• No-routine performed MRI • External “routinely” performed MRI • No data	62 2 0	
Indication for surgical treatment due		
• (Recurrent) Hemorrhage with new/progressive neurological symptoms • No data	64 0	
Follow-up time in years (mean ± SD)	8.1	7.4

### Clinical Management—Conservative and Operative Group

Conservative treatment was performed in 54 patients, while 64 patients underwent surgical resection of the BSCM (Figure 1). In the conservatively treated patient cohort, 17 patients (31.5%) stayed free of symptoms during FU, whereas 37 patients (68.5%) presented with neurological deficits. BSCM hemorrhage occurred in 41 (75.9%), whereas in 13 patients (24.1%) no BSCM hemorrhage was observed. During follow-up, an increase of size of the BSCM was radiologically confirmed in 15 patients (27.8%).

Among the 64 patients, 75 surgical procedures related to BSCM were performed in total, including 10 patients having undergone multiple surgeries. Two surgeries were performed due to the BSCM but without the aim of surgically resecting the BSCM (1 third ventriculocisternostomy, 1 external ventricular drain placement). In 9 instances, multiple surgeries were performed due to previously incompletely resected BSCM.

Surgery was performed within 3 months after initial diagnosis of BSCM in 38 patients, and at later stages of follow-up (>3 months) in 26 patients.

### Surgical Intervention Due to (Recurrent) BSCM Hemorrhage

Among the 64 patients that had undergone surgery, in 62 patients (96.8%) indication for surgical extirpation of the BSCM was done based on no-routine imaging that was performed in the context of clinical worsening (based on the KPS, mRS and NIHSS) as a direct consequence of BSCM (recurrent) hemorrhage.

In two patients, BSCM diagnosis was made and follow-up performed abroad. In both cases BSCM resection was not performed due to the lack of surgical experience at the institution where the patient was initially treated. Thus, surgery was deferred and in the patients were followed-up including routinely performed imaging studies. After continuous clinical deterioration due to the recurrent BSCM hemorrhage, the patients were ultimately referred to our Department of Neurosurgery where microsurgical removal of the BSCM was performed after the first consultation.

### Impact of Planned Follow-Up Imaging on Surgical Decision Making

During 961 follow-up years of 118 patients with BSCM, routinely performed follow-up MRI in clinically stable (= no clinical worsening based on the KPS, mRS and NIHSS) patients did not lead to a single indication for surgery.

## Discussion

The clinical course of patients with BSCM is difficult to predict and high quality evidence on follow-up management of BSCM is lacking (9). To date, there is no consensus on the best management for BSCM. The aim of this study was therefore to critically appraise the clinical management of patients with BSCM.

Currently, indication and timing of follow-up imaging in this patient population is highly individualized as relatively little convincing data is available to make evidence-based recommendations. Clinical management of patients with BSCM is thus largely based on the treating physician's subjective judgement, which aims at integrating relevant factors such as a patient's quality of life, size and location of the BSCM, previous hemorrhages and patient preference to make treatment recommendations. In the absence of indication for immediate surgical intervention, the two strategies most frequently pursued involve either active follow-up and serial imaging on a regular basis, or performing imaging studies only with new onset of suggestive symptoms such as atypical headache or new neurological deficit (8, 10). Independent of the specific approach recommended, patients should be treated at a neurosurgical referral center with experience in the clinical management of BSCM, given the rarity of BSCM and the complexity of its management (11).

Based on general recommendations for patients with CCM (8), we have typically performed routine follow-up imaging to monitor growth dynamics or asymptomatic hemorrhages in patients with BSCM at our institution. However, as there are no predictive models available for BSCM, it is unclear which radiographic changes predict a clinical course where immediate or early surgery is superior to conservative management with regard to clinical outcome. We hypothesized that, as a consequence, routine follow-up imaging will only lead to the decision to surgically treat the patient in very rare cases, if any.

In the cohort of 118 patients with BSCM treated at our institution a slight majority (54.2%) underwent surgery during a total of 961 years, whereas 46.8% were treated conservatively.

Of the 64 patients that were surgically treated for BSCM, in the overwhelming majority (96.8%) indication for surgery was done based on non-routine imaging that was performed in the context of new or progressive symptoms due to BSCM hemorrhage. Two patients, which were initially treated abroad, BSCM resection was not performed at the respective institution due to the lack of surgical experience. Ultimately, both patients underwent surgery after clinical deterioration and subsequent referral to our department. Therefore, during 961 follow-up years of 118 patients with BSCM, routinely performed follow-up MRI in clinically stable patients did not lead to a single indication for surgery.

The methodological limitations of retrospective and single-center cohorts, the heterogenous patient cohort and the lack of previously defined endpoints regarding clinical outcome represent significant limitations to this study. While the design of the present study does not allow for conclusions regarding the adequacy of the treatment decisions made, it can be clearly stated that the impact of routine follow-up imaging is very limited. These findings are in line with another study recently published by our group. There, we addressed the question whether routinely performed imaging had an impact on surgical decision making in patients with multiple CCM and found that it is highly questionable as there is no evidence for therapeutic relevance (12).

In general, it is likely a reasonable strategy to avoid imaging studies in absence of a clear indication. While the imaging process in and of itself can already cause discomfort and be inconvenient to patients, cost-effectiveness will become increasingly important in the context of rising healthcare costs and financial pressure. Avoiding relatively expensive imaging studies with unclear value regarding decision-making may be an attractive option to reduce healthcare spending without impairing the quality of patient care. However, a prospective study with a large number of patients is warranted to confirm our results.

Furthermore, although generally considered safe, repeated use of gadolinium-based contrast-enhancing agents has been linked to MRI signal changes and deposition of gadolinium in the brain (13). While the clinical significance of these deposits remains unclear, the fact that repeat contrast-enhanced imaging is indeed associated with measurable changes provides an additional rationale to minimize the number of MRI performed on a patient.

Another important aspect to consider is the impact of routine follow-up imaging on patients‘ psychology. It is conceivable that many patients may prefer not to be reminded of their diagnosis on a regular basis if there is no therapeutic consequence to the regular follow-up visits. On the other hand, it is possible that some patients would value the certainty of knowing on a regular basis that the CCM has not grown, especially those patients that had previously undergone routine follow-up imaging for many years. Regular follow-up consultation visits focusing on clinical evaluation without imaging studies could be a reasonable option to strike a balance between routinely performed imaging and no follow-up visits at all. In any case, careful patient education and inclusion of the patient in the decision process will allow the treating physician to individually tailor the treatment plan to the patient.

## Conclusion

Routinely performed imaging in clinically stable patients with brainstem cavernoma had no impact on surgical decision making in this series. Therefore, it appears reasonable to limit clinical management to patient education and symptom-driven follow-up strategy.

## Data Availability Statement

The datasets generated for this study are available on request to the corresponding author.

## Ethics Statement

This study was carried out in accordance with the recommendations of the University of Zurich. The protocol was approved by the Cantonal Ethics Committee (KEK-ZH; 2017-00330).

## Author Contributions

JV, FV, and OB: conception and design, acquisition of data, analysis and interpretation of data, and drafting the article. YY, MN, and LR: analysis and interpretation of data and critically revising the article. All authors contributed to the article and approved the submitted version.

## Conflict of Interest

The authors declare that the research was conducted in the absence of any commercial or financial relationships that could be construed as a potential conflict of interest.

## Abbreviations

BSCM: Brainstem cavernous malformations
CCM: cerebral cavernous malformations
cMRI: cerebral magnetic resonance imaging
CNS: central nervous system
KPS: Karnofsky Performance Scale
mRS: modified Rankin Scale
NIHSS: National Institutes of Health Stroke Scale.
